# Interactome-transcriptome analysis discovers signatures complementary to GWAS Loci of Type 2 Diabetes

**DOI:** 10.1038/srep35228

**Published:** 2016-10-18

**Authors:** Jing-Woei Li, Heung-Man Lee, Ying Wang, Amy Hin-Yan Tong, Kevin Y. Yip, Stephen Kwok-Wing Tsui, Si Lok, Risa Ozaki, Andrea O Luk, Alice P. S. Kong, Wing-Yee So, Ronald C. W. Ma, Juliana C. N. Chan, Ting-Fung Chan

**Affiliations:** 1 Hong Kong Bioinformatics Centre, School of Life Sciences, The Chinese University of Hong Kong, Shatin, N.T., Hong Kong; 2 Division of Genomics and Bioinformatics, School of Biomedical Sciences, The Chinese University of Hong Kong, Shatin, N.T., Hong Kong; 3 Department of Medicine and Therapeutics, The Chinese University of Hong Kong, Prince of Wales Hospital, Shatin, N.T., Hong Kong; 4 Li Ka Shing Institute of Health Science, The Chinese University of Hong Kong, Prince of Wales Hospital, Shatin, N.T., Hong Kong; 5 Hong Kong Institute of Diabetes and Obesity, The Chinese University of Hong Kong, Prince of Wales Hospital, Shatin, N.T., Hong Kong; 6 Department of Computer Science and Engineering, The Chinese University of Hong Kong, Shatin, N.T., Hong Kong

## Abstract

Protein interactions play significant roles in complex diseases. We analyzed peripheral blood mononuclear cells (PBMC) transcriptome using a multi-method strategy. We constructed a tissue-specific interactome (T2Di) and identified 420 molecular signatures associated with T2D-related comorbidity and symptoms, mainly implicated in inflammation, adipogenesis, protein phosphorylation and hormonal secretion. Apart from explaining the residual associations within the DIAbetes Genetics Replication And Meta-analysis (DIAGRAM) study, the T2Di signatures were enriched in pathogenic cell type-specific regulatory elements related to fetal development, immunity and expression quantitative trait loci (eQTL). The T2Di revealed a novel locus near a well-established GWAS loci *AChE*, in which *SRRT* interacts with *JAZF1*, a T2D-GWAS gene implicated *in* pancreatic function. The T2Di also included known anti-diabetic drug targets (e.g. *PPARD*, *MAOB*) and identified possible druggable targets (e.g. *NCOR2*, *PDGFR*). These T2Di signatures were validated by an independent computational method, and by expression data of pancreatic islet, muscle and liver with some of the signatures (*CEBPB*, *SREBF1*, *MLST8*, *SRF*, *SRRT* and *SLC12A9*) confirmed in PBMC from an independent cohort of 66 T2D and 66 control subjects. By combining prior knowledge and transcriptome analysis, we have constructed an interactome to explain the multi-layered regulatory pathways in T2D.

The majority of disease-associated variants discovered in Genome-wide Association Studies (GWAS) were located within non-coding regions of the human genome. These variants were significantly enriched in chromatin regulatory elements, particularly DNase I hypersensitive sites^1^, and expression quantitative trait loci (eQTL), suggesting these variants might influence disease risks by altering the expression levels of these disease-associated genes. Recent large scale eQTL studies including Multiple Tissue Human Expression Resource (MuTHER)^2^ and The Genotype-Tissue Expression (GTEx)^3^ provided expression data associated with genetic variants. These large datasets allowed identification of genes which might be regulated by these non-coding casual variants discovered in GWAS. In complex diseases, which often share similar traits, comorbidities and/or symptomatology, multiple disease genes interact to form overlapping genetic networks with interlinking pathobiological processes. Thus, the identification of a disease module may lead to discovery of other disease-related signatures in the vicinity of the gene-gene network^4^. To unravel this complexity, there has been a systematic effort to increase the coverage of the human interactome which is a map consisting of biologically relevant molecular interactions^5^. In this context, disorders that share same disease genes or connected by protein-protein interactions (PPI) often share comorbidity with overlapping symptomatology^6^,^7^. By combining clinical knowledge, human data, molecular interactions and cellular networks, we can explore correlations amongst these molecular/cellular networks and comorbidity patterns to generate new hypothesis on disease mechanisms.

Type 2 Diabetes (T2D) is a model of complex multi-system disease involving the brain, gut, liver, pancreas, muscle, fat and kidney^8^. Compared to non-diabetic subjects, people with T2D have 1.3–3 fold increased risk of vascular, cancer, non-vascular (renal, neurological, gastrointestinal, hepatic, sepsis) and non-cancer deaths^9^. In this experiment, we used high-throughput transcriptome sequencing (RNA-Seq) to investigate gene expression using peripheral blood mononuclear cells (PBMC) in a case-control cohort of Chinese T2D patients and age- and gender-matched healthy subjects. Based on the premises that (1) disease-associated proteins tend to cluster in the interactome to form disease modules^10^, (2) disease-associated genes show distinct interaction patterns in the interactome^11^ and (3) these interaction patterns may influence expression patterns, we used complementary strategies to identify disease-associated genes in an interactome^12^. Specifically, we used the Subnetwork-based method which identifies highly-connected modules within the interactome and the Diffusion-based method which identifies candidate genes connected to known disease genes in the interactome. In this study, we applied these two strategies in an integrated transcriptome-interactome analysis to enhance our understanding of the pathogenesis of T2D.

## Results

### Study overview

**Figure 1:**
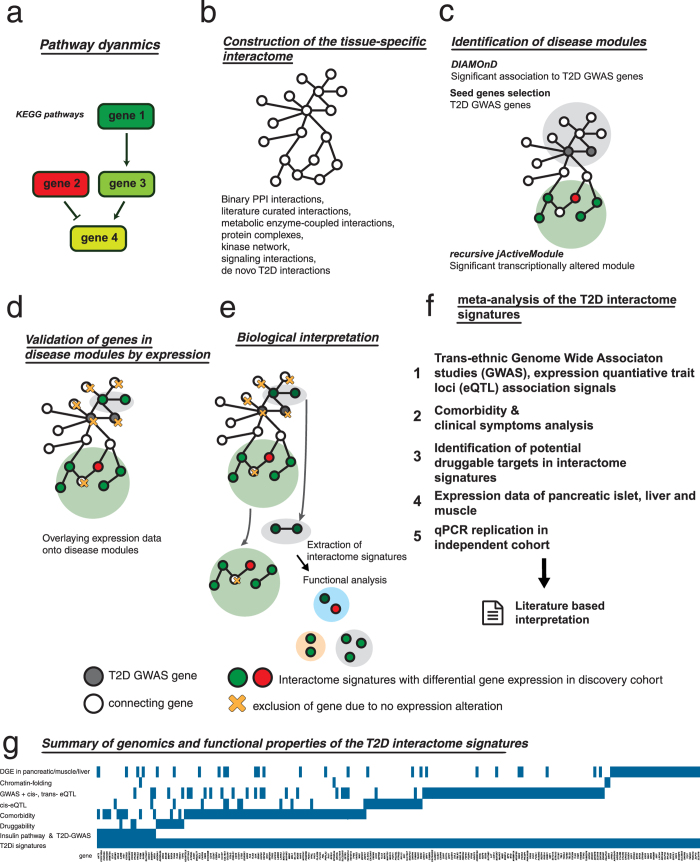
Overview of the integrated T2D study design. (**a**) Analysis of pathway dynamics in T2D. (**b**) T2D interactome was constructed from curation of known interactions and T2D co-expression patterns. (**c**) Disease modules were identified through identification of genes significantly associated with T2D-GWAS loci, and gene clusters which were significantly altered transcriptionally in the discovery case-control cohort. (**d**) The genes in the disease modules were filtered and validated based on differential gene expression in our dataset which yielded the final interactome signatures. (**e**) The resultant interactome signatures were interpreted using a functional network. (**f**) The interactome signatures were validated through comparative analysis with DIAGRAM GWAS, eQTL studies including MuTHER and GTEx, trait and druggability analysis, expression in pancreatic islet, liver and muscle, followed by qPCR replication of genes in an independent case-control cohort. Refer to respective section for details. (**g**) T2D interactome signatures overlapping with various genomic and functional properties are defined as follows: “DGE in T2D pancreatic/muscle/liver” indicates the signatures were also dysregulated in T2D relevant tissues, in addition to our discovery cohort; “Chromatin-folding” indicates the genes which may be distantly regulated by the GWAS SNPs reaching genome-wide significance in DIAGRAM-database; “GWAS + cis-, trans-eQTL” refers to the genes identified by the Sherlock statistical framework to be associated with T2D using the DIAGRAM-database, cis- and trans- eQTL signals; “cis-eQTL” refers to the SNPs in perfect LD to T2D interactome signatures with eQTL properties regulating these genes; “Comorbidity” refers to genes that are shared between T2D and comorbid diseases; “Druggability” indicates T2D druggable or potentially druggable targets; “Insulin & T2D-GWAS” refers to genes in the insulin pathway and T2D genes in GWAS Catalogue with dysregulation in the T2D interactome signatures.

Accumulating evidence suggested that peripheral blood is a useful surrogate tissue for discovery of disease signatures as gene expression levels in blood cells may reflect metabolic phenotypes^13^. We performed a cross-sectional case-control study of RNA expression in PBMC using high-throughput strand-specific transcriptome sequencing. Figure 1 summarizes the study design. Strand-specific transcriptome sequencing for 10 T2D individuals and 10 controls generated an average of 5.2Gb sequencing data per individual (Table S1). We first analyzed the changes in KEGG pathway dynamics in T2D (Fig. 1a). In line with previous findings^14^,^15^, we observed modest changes in gene expression with up-regulation of inflammatory and immunity-related genes. We also observed inhibition of insulin signaling and confirmed altered expression of some T2D-GWAS genes in PBMC (see section “Differential gene expression and alteration of pathway dynamics”). Then, we reconstructed the T2D interactome (T2Di) (Fig. 1b) by combining 1) genes significantly connected to T2D-GWAS loci in the interactome and 2) gene clusters with significant changes in transcription (Fig. 1c,d) to identify novel molecular signatures associated with T2D. The functional and pathobiological relevance of the disease module was validated computationally in our case-control cohort (Fig. 1e). We selected a sample of candidate genes from the modules in T2Di and used qPCR to replicate their expression in PBMC from an independent case-cohort cohort (Fig. 1f). The rationale and methods of construction of the interactome, identification of novel T2Di signatures, choices of genes subjected to qPCR validation, and various meta-analyses (Fig. 1f) are discussed below. A summary of the genomic and functional properties of these T2Di signatures are shown in Fig. 1g and Fig. S1.

### Incomplete human interactome necessitates the construction of a T2D interactome (T2Di)

In the human interactome map constructed by Menche *et al*. using physical protein interactions supported by experimental validation^5^, the largest size (S) of the T2D module, as defined by Online Mendelian Inheritance (OMIM) and GWAS was 9 (S = 9), i.e. the module consisted of 9 T2D-GWAS genes which physically interact with each other. This was larger than random expectation S^rand^: 2 ± 1 (z score: 3.9, *p*: 4.80E-5) suggesting that the observed T2D module was unlikely to be due to random agglomeration of any disease genes. Since there were 97 T2D-GWAS genes, the relative module size (S/Nd), where Nd was the number of known T2D-associated genes was only 9.3% (9/97) (Fig. S2). This relatively low module size was likely due to incompleteness of the currently available human interactome^5^. Apart from the module size, the degree of clustering and distance between proteins also gave some indications regarding the completeness of the interactome. In Menche’s module, disease proteins not connected to T2D-associated genes tended to cluster in the neighborhood (*p*: 1.20E-2). The network-based distance amongst these possibly disease-related proteins in juxtaposition (*d*_*s*_) was large with small effect size (Glass’ Delta) (Fig. S3). These data indicated that the current human interactome required enhancement for discovery of T2D modules.

In this analysis, we combined known interactions and our *de novo* T2D co-expression network to construct an interactome comprising 19,984 genes (nodes) connected through 455,302 interactions (edges). The degree distribution of our interactome followed a power law (Fig. S4) and approximated a scale-free network, a property observed in many biological networks. Mathematically, it has been estimated that there are 650,000 interactions in a human interactome^16^. By integrating the interactome and transcriptome analysis, our T2Di contained approximately 70% of the human interactions. Note that the *de novo* network constructed by ARACHE constituted only a minority (6%) of the interactions in the T2Di (Fig. S5).

### Differential gene expression and alteration of pathway dynamics

In the transcriptome analysis, using a criterion of absolute log2 fold-change >0.75, 157 genes were significantly up-regulated and 1,238 genes were significantly down-regulated in T2D (Table S2). The magnitude of global change in expression was modest (T2D up-regulated: median log2fold 0.81; T2D down-regulated: median log2fold −1.23).

In the T2D subjects, expression of 11 T2D-GWAS candidate genes (*IRS1*, *WFS1*, *KCNQ1*, *CHCHD9*, *CDKN2A*, *SLC16A11*, *GPSM1*, *CCDC102A*, *IL2RB*, *PPARD*, *DNMT3A*) were repressed in their PBMC (Table S3). In order to provide a generalized overview of our case-control cohort, we performed standardized analyses including gene ontology and KEGG pathway dynamics to identify functional gene enrichment as well as pathway activation and repression.

In animal studies, adaptive and innate inflammatory responses in visceral adipose tissues due to accumulation of B cells, activation of pro-inflammatory macrophages and production of pathogenic IgG antibodies by T cells may mediate insulin resistance and glucose intolerance^17^. These maladaptive immune responses supported the use of expression signatures of PBMC as immune cells to study mechanism of T2D and insulin resistance. In T2D patients, we identified down-regulated genes in the insulin-signaling pathway (Table S3), such as *AKT2* (Log2fold: −0.78, adjusted *p*: 4.42E-4). In experimental studies, silencing *AKT2* resulted in inhibition of insulin-mediated glucose uptake and glycogen synthesis^18^. In addition to insulin receptor substrate 1 (*IRS1*), (log2fold −0.98, adjusted *p*: 3.93E-3), other down-regulated genes were enriched in “signal transduction” of the MAPK and JNK cascade (adjusted *p*: 5.45E-3) and “secretion” of hormone activity (adjusted *p*: 1.96E-2). The up-regulated genes were enriched in the Gene Ontology inflammation-related term “defense response” (adjusted *p*: 2.02E-2) which included genes such as *CAMP*, *TLRs*, *S100A12* and *SELP* (Table S4). We also analyzed changes in gene expression using SPIA^19^ which takes into consideration quantitative changes in expression and gene-gene interactions within the context of pathway topology. Using this approach, we discovered repression of “Wnt signaling pathway” (adjusted *p*: 2.40E-02) and “Dopaminergic synapse” (adjusted *p*: 1.50E-02) in T2D (Table S5). In this light, D2-Dopamine agonist which restores hypothalamic dopamine level has been approved by the U.S. Food and Drug Administration (FDA) for treatment of T2D^20^. Apart from the “Cytokine-cytokine receptor interaction” pathway (adjusted *p*: 2.00E-03) and “Chemokine signaling pathway” (adjusted *p*: 2.70E-02), the “Circadian rhythm” (adjusted *p*: 5.00E-06) was also activated in T2D, in line with the association of dysregulation of circadian clocks with metabolic diseases^21^.

### Interactome signatures reflect pathogenesis and druggability of T2D

**Figure 2:**
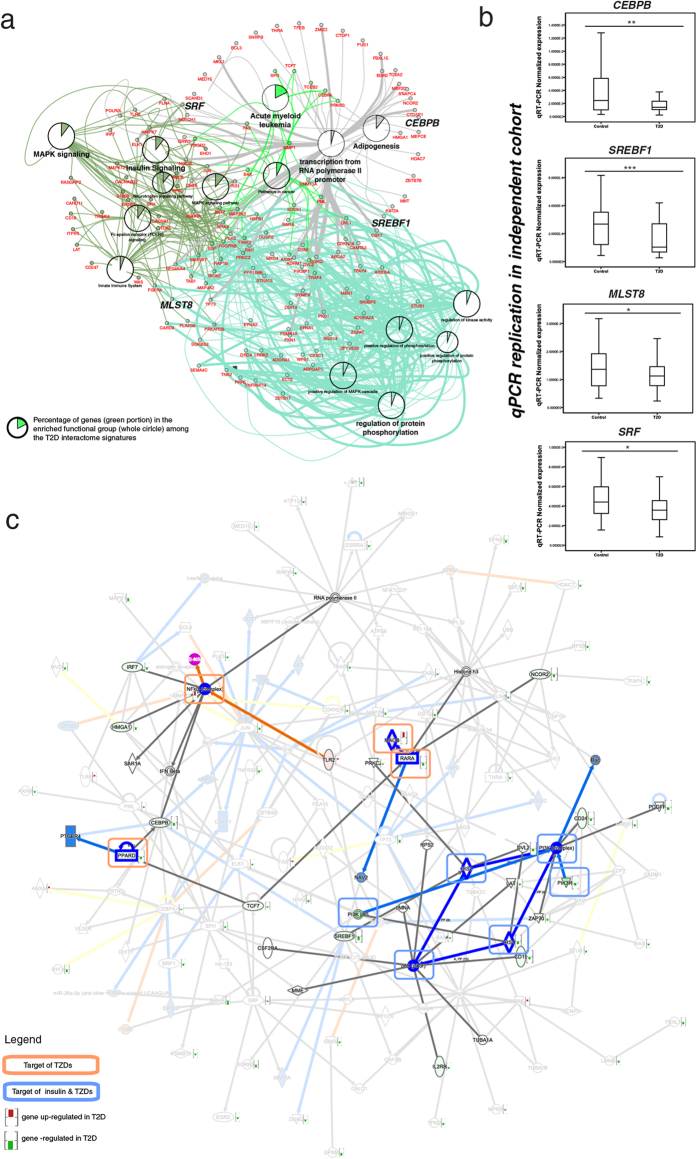
Functional analysis of the T2D Interactome Signatures. (**a**) Functional analysis of the T2D interactome signatures revealed enrichment of genes related to insulin signaling, MAPK signaling, acute myeloid leukemia, transcription, adipogenesis and regulation of protein phosphorylation. Color(s) of the nodes and the line connected to the functional grouping(s) indicate the function(s) of the respective gene. The thickness of the edge represents the evidence code of the Gene Ontology that relates the gene (node) to the functional term. The thicker edge represents those with experimental evidence code. (**b**) The dysregulation of expression of *SREBF1*, *CEBPB*, *MLST8* and *SRF* was replicated in the independent cohort. Statistical significance in change of gene expression: *p < 0.05, **p < 0.01, ***p < 0.001. (**c**) T2D Interactome signatures targeted by anti-diabetic medications.

In this integrated human interactome with connections to T2D-GWAS genes and our *de novo* transcriptome data, we identified 420 Interactome signatures (T2Di signatures) supported by transcriptional dysregulation in PBMC of our case-control cohort (Table S6). Using computational analysis, we validated the biological significance of these signatures, which were enriched with gene functions in “insulin signaling”, “MAPK signaling”, “acute myeloid leukemia”, “transcription”, “adipogenesis” and “regulation of protein phosphorylation” (Fig. 2a). In T2D, defective adipocyte differentiation results in ectopic fat distribution with excessive calorie intake^22^. The transcription factor, PPAR-G, promotes adipogenesis with CEBPB as the upstream signal. Insulin signaling activates mTOR to increase hepatic gene expression through the mTORC2 complex, and lipogenesis by modulating gene expression of *SREBF1*. The formation of mTORC2 complex is dependent on *MLST8* (or rictor) expression and disruption of mTORC2 by silencing *MLST8* can lead to insulin resistance in rodent model^23^. Glucose binds to the nuclear receptor LXRbeta promoter^24^ to regulate energy utilization in muscle and fat storage in visceral adipose tissue^25^. Here, SRF regulates glucose binding to LXRbeta with silencing of *SRF* resulting in impaired glucose-mediated cellular responses^24^. Given the crosstalks amongst insulin, *IGF-1* (insulin growth factor-1) and *SREBF* pathways, reduced *SREBF1* expression has been reported in obesity and T2D in adipose tissue^26^. In support of these disease mechanisms, we found repressed expression of *CEBPB*, *MLST8*, *SREBF1* and *SRF* in our discovery cohort with validation in the replication cohort (Fig. 2b).

Some of the molecular signatures of the T2Di overlapped with gene targets of FDA-approved anti-diabetes drugs. For example, *NF-kB*, *PPARD*, *MAOB and RARA are* downstream targets of Thiazolidinedione (TZDs) while *P1K3 pathway*, *IRS1 and IRS2* are that of insulin^27^,^28^. In the vicinity of these drug targets (first degree connection), two of the T2Di signatures, namely *NCOR2*^29^ and *PDGFR*^30^, are potential T2D drug targets (Fig. 2c). Using the Drug Gene Interaction Database and manual literature curation, we identified possible druggable targets including *ADORA1*/*2*, *DRD4*, *IL2RB*, *THRA*, *TNNC1*, *TSPO*, *MMP1* and *TLR8* with supporting evidence summarized in Table S7.

### Interactome signatures supported by disease comorbidity and clinical symptoms

The maintenance of human structure and function is regulated by interlinking biological systems. Internal (e.g. aging) and external causes (e.g. environment and lifestyle) can disrupt these processes leading to cascades of dysfunctions to trigger co-emergence of multiple diseases. In system biology, there are significant correlations amongst the molecular structures of disease modules and cellular networks associated with multiple diseases and morbidities. Diseases that share genes, PPI and pathobiological pathways tend to exhibit overlapping symptoms and comorbidity^6^,^31^. Recent reports indicated that diseases with similar symptoms, as quantified by MeSH metadata using ‘term frequency–inverse document frequency’, tend to share disease genes with high first- and second-order PPI amongst the disease-related proteins^7^. Due to their frequent clustering, we expected the T2Di signatures to be enriched in genes related to T2D associated comorbidities and symptomatology.

To test this hypothesis, we first obtained the T2D-associated genes by mapping the disease genes from OMIM and GWAS to diseases according to the method described by Menche *et al*.^5^. According to the U.S. Medicare disease history of more than 31 million individuals, T2D patients had a relative risk ≥1.5 (99% confidence interval >1.0) increased for 70 diseases (ICD-9 codes)^32^, Comorbidity analysis of these disease-associated genes indicated that the T2Di signatures were enriched with genes associated with 27 comorbid diseases, including male urogenital diseases^33^. (RR: 1.86, enrichment *p*: 3.18E-2, Fisher’s exact test), gastroenteritis (RR: 1.51, enrichment *p*: 1.77E-3, Fisher’s exact test) and cardiomyopathy (RR: 1.74, enrichment *p*: 3.66E-3, Fisher’s exact test)^34^. Using the ‘diseases symptoms similarity’ captured by large-scale medical bibliographic record^7^, there were also high similarities between T2D and atherosclerosis (similarity score: 0.74) and male infertility (similarity score: 0.45). The correlations of these comorbid diseases and symptomatology within the context of PPI with enrichment of shared genomic signatures supported the validity of our T2Di signatures (Table S8).

### Partial explanation of residual associations with DIAGRAM by T2Di signatures

**Figure 3:**
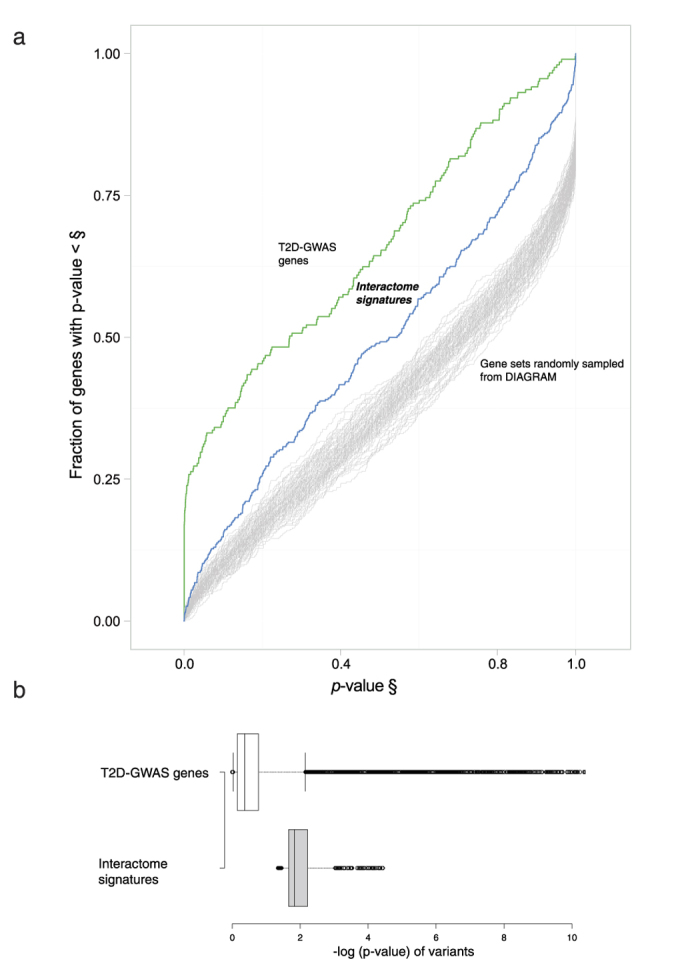
Comparison of T2Di signatures with known T2D GWAS genes. (**a**) Comparison of enrichment of different gene sets using the DIAGRAM meta-analysis dataset. The T2D-GWAS genes obtained from GWAS Catalog has the highest enrichment, followed by our T2Di signatures. The 100 random control gene sets (size matched to the Interactome Signature set) randomly sampled from the DIAGRAM dataset have significantly lower fractions of low *p*-value genes. (**b**) Comparison of DIAGRAM *p*-values of the variants of T2D-GWAS genes with T2Di signatures.

We used Quantile-Quantile (QQ) plot to study all associations in the DIAGRAM-database (black curve in Fig. S6) and found residual associations after removing the T2D-GWAS SNPs and SNPs in linkage disequilibrium (LD) (green curve), suggesting many T2D loci are yet to be discovered in the DIAGRAM database. Using GWAS as the gold standard for discovery of causal genes, we used the DIAGRAM meta-analysis database to test whether the T2Di signatures (without established T2D GWAS genes) were enriched with variants associated with T2D, which fell short of genome-wide significance. We first computed a single *p-*value for each gene in the DIAGRAM-GWAS using the LDsnpR method^35^. Figure 3a shows the distribution of the combined *p-*value of T2D-GWAS genes (from 46 T2D GWAS datasets), T2Di signatures, and 100 control gene sets randomly sampled from the DIAGRAM data with a size matched to the T2Di signature set. The result indicated that previous T2D-GWAS contained the highest fraction of T2D-associated genes in the DIAGRAM dataset, followed by the T2Di signatures and finally the control sets. This observation suggested that our T2Di signatures were enriched with low frequency disease-susceptibility variants not expected by chance. In order to prove the utility of combining physical interaction network and co-expression network, we applied the two module identification algorithms on physical and co-expression network independently and cross-compared the potential disease genes based on DIAGRAM GWAS with the integrated discovery method. The physical interaction network contains 18696 genes with 424550 interactions, whereas the co-expression network contains 6316 genes with 30965 interactions. jActiveModules identified fractions of genes with more significant *p*-values in an integrated interactome than in annotated physical network and co-expression network. Similar observation was observed for DIAMoND algorithm, such that DIAMoND applied on an integrated interactome discovered a higher fraction of genes with more significant *p*-values than in annotated physical network and co-expression network (Fig. S7). Taken together, we have shown that by merging physical interaction network and co-expression network as an integrated T2D interactome, a higher fraction of genes with more significant GWAS association to Type 2 diabetes could be discovered.

The advantage in combining Diffusion-based method (DIAMoND) and subnetwork-based method (e.g. jActiveModules) could be observed by dividing the T2Di signatures into 2 groups: (1) the gene sets discovered by DIAMoND and (2) the gene set discovered by jActiveModules. As shown by Fig. S8, the differential expressed genes (DGE) obtained in this study (i.e. without undergoing any selection by interactome analysis) were enriched in genes with more significant *p*-values than random gene sets. DIAMoND and jActiveModules both contained genes with *p*-values more significant than DGE gene set, suggesting the use of both methods independently further enriched T2D relevant genes.

Because DIAMoND and jActiveModules identified different set of genes (DIAMoND identified 39 genes whereas jActiveModules identified 401 molecular signatures; both method identified 20 common genes; Table S6), the functional cohesiveness among genes discovered by DIAMoND and jActiveModules in T2Di signatures could first been demonstrated in the similarity of pathways enriched from respective methods (Fig. S9). The functional, genomic and genetic relevancy of the T2Di signatures to Type 2 diabetes is demonstrated in the enrichment of such properties of DIAGRAM SNPs in T2D interactome signatures over random, as described in next section.

Figure 3b shows the distribution of variants’ *p-*values of T2D-GWAS genes versus our T2Di signatures. Since the latter did not reach the nominal genome-wide significance (*p*: 5.00E-8), the T2Di signatures could not be discovered through GWAS alone.

### Genomic and genetic properties of DIAGRAM SNPs in T2D interactome signatures

Many GWAS SNPs are located within transcriptional regulatory regions, including promoters and enhancers, which are enriched with eQTL property^36^. Therefore, we expected genuine T2Di signatures to possess properties similar to T2D-GWAS genes. In line with such notion, we found a significant fraction (49.4%; 270/547) of DIAGRAM genome-wide significant SNPs (*p* < 5.00E-8) overlaps with regulatory regions [transcription factor (TF) binding, DNase I hypersensitivity peak/footprint, sequence motif, eQTL or a combination of them]. We performed 1 million times of Monte Carlo randomization of a size-matched SNP set from DIAGRAM-database and found that the T2D-GWAS variants with genome-wide significance were enriched in regulatory elements (*p*: 1.00E-06). Similarly, SNPs in perfect LD with the T2D-GWAS variants were also enriched in regulatory elements (*p*: 3.20E-3). Notably, we also observed enrichment of SNPs in perfect LD with our T2Di signatures (75.6%; 875/1157, *p*: 1.00E-06) which overlapped with regulatory elements.

**Figure 4:**
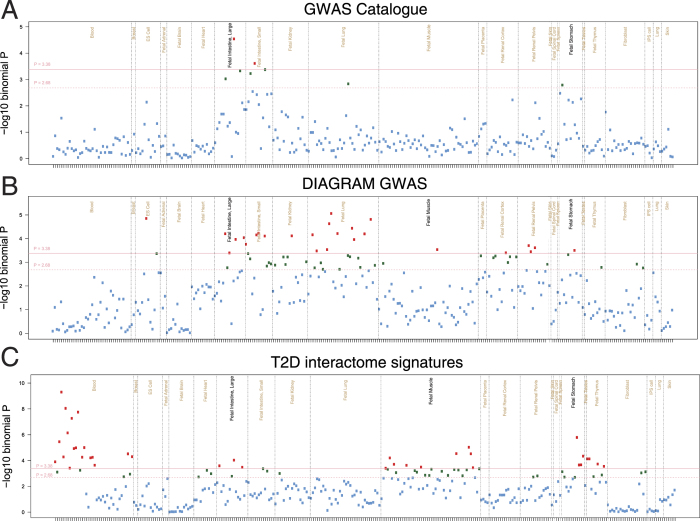
Enrichment of SNPs on DNase I hotspots. Each point represents the enrichment of the test SNP set compared to matched background SNPs on a single sample, organized by tissue types. (**A**) Type 2 Diabetes SNPs in GWAS Catalogue (**B**) DIAGRAM SNPs meeting genome wide significance of *p*: 5.00E-8. (**C**) SNPs associated with the T2Di signatures in the DIAGRAM dataset. Red points are at adjusted *p* ≤ 0.01, and green points are at *p* ≤ 0.05.

Since many disease-associated GWAS variants are enriched in affected cell type-specific regulatory elements^1^, we sought to identify tissue-specific enrichment of regulatory elements in SNPs that were in perfect LD with the T2Di signatures. We found that the established T2D-associated variants (in both T2D-GWAS and DIAGRAM) satisfying genome-wide significance were enriched in fetal regulatory elements (Fig. 4A,B), including fetal intestine, muscle and stomach. The enrichment of GWAS variants in fetal regulatory elements is commonly seen in complex diseases for which growth trajectory plays important pathogenetic roles^1^. Similarly, the SNPs in perfect LD with our T2Di signatures were also enriched in regulatory variants in fetal intestine, muscle and stomach, in addition to blood and thymus. In T2D, dysregulation of auto-immunity has been reported and in db/db mice, thymus transplantation restored cytokine imbalance and insulin sensitivity^37^.

Among the 420 interactome signatures, 43.3% (182/420) overlapped with TF binding sites together with DNase I peaks. Amongst them, 17.6% (32/182) were rSNP/eQTL signals with genome-wide significance (*p*: 5.00E-8) in DIAGRAM or in perfect LD to these DIAGRAM SNPs. These SNPs were associated with expression changes in GTEx (skeletal muscle, whole blood and subcutaneous Fat) and MuTHER (lymphoblastoid cell lines (LCL) and fat). The T2Di signatures in perfect LD to DIAGRAM variants with *p*-values between 7.60E-5 and 3.60E-2 (Table S9) were also enriched in rSNP/eQTL property (Fisher *p*: 3.50E-2; compared to a matched set of random sampling of 182 SNPs having exact range of GWAS *p*-value). Of note, the *p*-values of variants of genes targeted by anti-diabetic drugs (with the exception of *IDE*, *PPARG*, *KCNJ11* and *ABCC8*, which are established T2D loci) were between 4.28E-5 and 6.75E-3 in the DIAGRAMv3 GWAS, not reaching genome-wide significance^27^. Our T2Di signatures possessed many significant eQTL loci (Fig. S10A) including those expressed in LCL (Fig. S10B) and fat tissue (Fig. S10C) although only variants associated with *JAZF1-AS1* and *WFS1* reached genome-wide significance (*p* < 5.00E-8).

Finally, we combined *cis*- and *trans*- eQTLs and GWAS *p*-values to assess our T2Di signatures. Using the Sherlock statistical framework^38^, we identified 17 (24.3%; 17/70) DIAGRAM-genes to be associated with T2D (*p* < 0.05) (Fisher *p*-value for enrichment: 3.32-5) compared to enrichment of 88 genes in the T2Di signatures with *p* < 0.05 (20.9%; 88/420) (Fisher *p*-value for enrichment: 5.40E-14) (Table S10).

### Differential expression of SRRT and SLC12A9 led to discovery of novel locus near AChE

**Figure 5:**
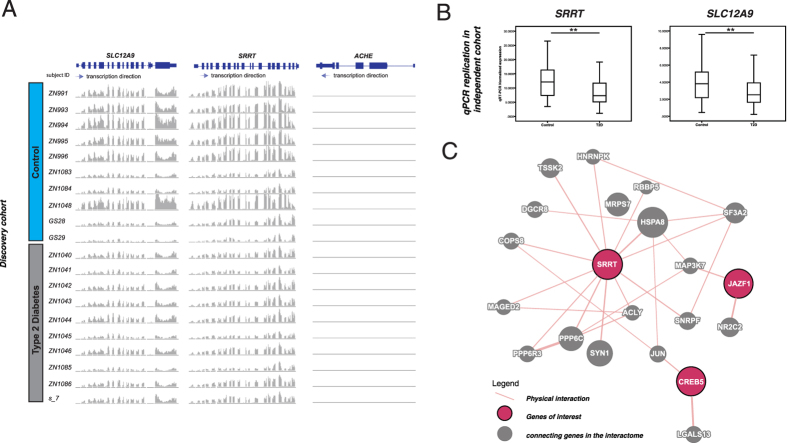
GWAS significant T2D association of SNP rs7636 might also be functionally explained by repression of *SRRT* and *SLC12A9*. (**A**) rs7636 is in perfect LD with rs11171 (*SRRT*), rs781190 and rs1716255 (*SLC12A9*) in Beijing Han Chinese in the CSHL-HapMap project (r2: 1; D’:1). Both *SRRT* (log2fold -0.88, adjusted *p*: 1.24E-4) and *SLC12A9* (log2fold -0.77, adjusted *p*: 6.32E-3) were significantly down-regulated in T2D. Expression of *ACHE* was not altered (log2fold 0.19, adjusted *p*: 1.00). (**B**) The dysregulation of expression of *SRRT* and *SLC12A9* were replicated in the independent cohort. Statistical significance in change of gene expression: * *p* < 0.05, **p < 0.01, ***p < 0.001. (**C**) Physical interaction of *SRRT*, *JAZF1* and *CREB5* among other proteins.

In the Asian GWAS datasets, we noted that *rs7636*, a synonymous SNP within Acetylcholinesterase (*AChE*), exhibited genome-wide significance for T2D in Asian but not Caucasian populations with an odd ratios of 1.85 (95% CI: 1.42–2.41, *p*: 5.00E-6)^39^. This SNP was in perfect *LD* with multiple SNPs which might regulate expression of two T2Di signatures, *SLC12A9 and SRRT* (Table S11), which are in proximity with each other but distant from *rs7636* (Fig. 5A). Both *SRRT and SLC12A9* were down-regulated in T2D (*SRRT*: log2fold -0.87; adjusted *p*: 4.46E-5; *SLC12A9* log2fold: -0.76; adjusted *p*: 5.87E-4) with replication in our independent cohort (Fig. 5B). According to the GTEx analysis V4 (dbGaP Accession phs000424.v4.p1), *AChE* is mainly expressed in brain (median RPKM: 10.74; 357 samples) with low expression in blood tissues (median RPKM: 0.73; 245 samples).

GWAS variants located in regulatory regions may control distant genes through long-range interactions^1^. We used chromosome conformation capture (Hi-C) data to test this hypothesis. Using two independent normalization methods, HiCNorm^40^ and Yaffe^41^ to identify consensus interacting partners in K562 and GM06990 cell lines, we identified the chromatin region containing rs1635852 and other DIAGRAM-SNPs reaching genome-wide significance that might interact with *SRRT* by a long-range interaction (Table S12). In particular, rs1635852 was associated with *CREB5* and *JAZF1* expression. The latter is an established T2D-GWAS locus^42^ which is a transcriptional repressor of gluconeogenic genes^43^ and regulator of visfatin expression via PPARα, and PPARβ/δ signaling^44^. In T2D islets, the risk allele rs1635852-T of *JAZF1* was associated with lower transcription enhancer activity^45^. In our T2Di, *SRRT*, *JAZF1* and *CREB5* physically interact, along with other proteins (Fig. 5C). While *JAZF1* and *CREB5* bind to the miRNA miR-9, which has been implicated in pancreatic development, *JAZF1* also binds to miR-96 that negatively regulates insulin exocytosis^46^. In this connection, *SRRT* contributes to the stability and delivery of capped primary miRNA transcripts to the primary miRNA processing complex and may modulate expression through gene silencing. Together with the genome-wide significance of *rs7636* in Asian populations, our analysis revealed a possible network linking *SRRT and JAZF1* possibly through miRNA.

### Assessment of T2Di signatures by an orthogonal approach

Recently, Himmelstein *et al*. developed a computational method to prioritize and predict disease-associated genes^47^. In their method, GWAS genes were used as gold standard for assessing the prediction accuracy. Firstly, features that describe the topology between diseases and the known GWAS-associated genes were extracted and analyzed in a metagraph network constructed using diverse information domains. Then, PathPredict, a machine learning algorithm originally developed for social network analysis, was used to train a model from these GWAS associations, and then applied to other protein-coding genes to predict the probability of disease associations. Their Heterogeneous Network Edge Prediction method, based on abovementioned principles, which is distinct from our multimethod interactome approach to discover disease-related genes, can be used to evaluate our T2Di signatures. We used the probability scores computed by Himmelstein *et al*. to compare our T2Di signatures, segregated by overlapping with genomic and functional properties, to the established GWAS-T2D genes using randomized, size-matched gene sets (Fig. S1). We found that our T2Di signatures had median probability scores similar to those of T2D-GWAS loci that were higher than expected by chance (Fig. S11) and provided further support to our T2Di signatures.

## Discussion

Common human diseases such as diabetes, are due to complex interactions of many genes, each with small effect size^48^. While GWAS has led to the discovery of multiple disease-associated genes or SNPs, pathway analysis can provide further insights into the interactive effects of these genes/SNPs in disease development. In this integrated transcriptome-interactome analysis, we employed various strategies utilizing prior knowledge such as the T2D-GWAS loci to analyze the transcriptome of PBMC in a case-control cohort. Firstly, gene ontology, pathway enrichments and pathway dynamics analysis were performed to provide a general overview of the case-control cohort. We performed functional Over-Representation-Analysis (ORA) to identify a set of differentially-expressed genes (DEGs), followed by enrichment analysis of these DEGs in known biological pathways and Gene Ontology terms. Although we were able to demonstrate perturbation of T2D-associated pathways using this method, the ORA approach only took into consideration the number of genes, assuming each gene being independent and ignored their expression changes. We then used pathway topology and took expression changes into account to infer the pathway dynamics although there remained challenges due to limited number and incomplete coverage of annotated human pathways^49^.

Based on the premise that complex diseases are caused by a combination of molecular perturbations^50^ to form disease modules^5^ that tend to avoid hub genes which might cause major abnormality^51^, we integrated our gene expression analysis within the context of a human interactome. Using various methodologies, we identified novel interactome signatures shared by diseases with comorbidity and symptomatology related to T2D. We note that our approach to discover novel candidate genes share similar concept with Sharma *et al*.^52^, where multiple methods, including jActiveModules, were combined to discover disease genes. Our approach was based on distinct premise that disease genes could be discovered by transcriptome-interactome analysis, in which clusters of transcriptionally altered gene signatures and disease genes with significant fractions of their connections to T2D-GWAS genes were discovered by jActiveModules and DIAMoND, respectively, followed by experimentally validation by RNA-Seq of our case-control cohort. These signatures are enriched in regulatory elements, particularly cell-type specific regulatory elements, notably fetal intestine, stomach and muscle. Apart from explaining some residual associations in the DIAGRAM GWAS, some of these signals also showed associations with eQTL in human tissues. We further identified an Asian-relevant T2D locus, *AChE*, upstream of *SRRT*, the latter being differentially expressed with physical interaction with *JAZF1*, a T2D-GWAS gene implicated in beta cell biology, possibly through long distance chromatin regulation and miRNA.

## Conclusion

By integrating prior knowledge with transcriptome and interactome analysis, we discovered disease modules and druggable pathways to explain the complexity of diabetes. In this post-GWAS era where multiomic technologies are increasingly used to study gene regulation and expression, our multi-method approach can be used to provide new insights into the pathogenesis of complex diseases.

## Materials and Methods

The methods and results of the supplementary datasets are explained in the corresponding files.

### Ethics, consent and permissions

Written informed consent was obtained from all participants. This study was approved by the Clinical Research Ethics Committee of the Chinese University of Hong Kong. All experiments were performed in accordance with relevant guidelines and regulations.

### Subjects of discovery and replication cohort and RNA sequencing

All participants in this study were of southern Han Chinese ancestry residing in Hong Kong. All T2D cases were selected from the Hong Kong Diabetes Registry and from the Hong Kong Family Diabetes Study. Type 2 diabetes was diagnosed according to the 1998 World Health Organization criteria. Patients with type 1 diabetes defined as acute ketotic presentation or continuous requirement of insulin within 1 year of diagnosis were excluded. In our discovery cohort, we selected 10 T2D patients (50% male, age 52.8 ± 12.3 years, disease duration: 22.6 ± 11.8 years) with enriched phenotypes including positive family history, early age of diagnosis, and obesity (age of diagnosis: 30.2 ± 8.1 years; body mass index (BMI): 29.9 ± 4.7 kg/m^2^)^53^. The replication cohort included 66 T2D individuals and 67 healthy controls. All control subjects were age- and gender-matched with fasting plasma glucose below 6.1 mmol/l who were either hospital staff or volunteers from a community-based health screening program. The clinical phenotypes of the discovery and replication cohort are shown in Tables S13 and S14, respectively.

Type 2 diabetes is characterized by inflammatory infiltrates in pancreatic islets and accumulation of T-cell in adipose tissues^54^. Human studies supported alterations of gene expression in peripheral blood, which provided a glimpse into the internal environment^13^. Islet-infiltrating immune cells were in equilibrium with circulating pools, which could be sampled via peripheral blood. Thus, we hypothesized that changes in gene expression in PBMC might provide insights into the molecular mechanisms underlying the metabolic dysregulation in T2D. By sampling PBMC in T2D and control subjects, we systematically characterized the transcriptome to discover molecular networks and signatures associated with T2D-GWAS genes. We first isolated PBMC from blood using a Ficoll gradient followed by extraction of total RNA using Trizol reagent. Poly(A)^+^ RNA was purified with the Dynabeads mRNA purification Kit (Life Technologies) following the manufacturer’s instructions and then subjected to dUTP-based strand-specific RNA transcriptome sequencing on the Illumina GAIIx platform.

### Sequence and pathway analysis

Sequencing reads were dynamically trimmed according to BWA’s algorithm with parameter -q 20. Read pairs were synchronized such that all read-pairs with sequences equal to or longer than 35 bp on both sides after trimming were retained^55^. Quality trimmed reads were mapped onto human genome (hg19) by STAR v.2.3.0e^56^ and gene expression in terms of FPKM was calculated by Cufflinks v.2.2.1^57^. Differential gene expression was analyzed using DESeq2^58^, and genes with Benjamini and Hochberg multiple-testing adjusted *p* < 0.05 and absolute log2 fold-change >0.75 were considered to be significant. KEGG Pathway dynamics analysis was performed using the Signaling Pathway Impact Analysis (SPIA)^19^. Over-Representation-Analysis: Gene ontology enrichment analysis was performed using BiNGO v.2.44^59^ implemented in Cytoscape v.2.82 (http://cytoscape.org/). Enrichment was considered to be significant when *p*-value was less than 0.05 after multiple-testing adjustment with the Benjamini and Hochberg procedure. Expression data from mammalian pancreatic islet^60^, β-cell^61^, liver^62^ and skeletal muscle^63^ were retrieved from NCBI GEO (http://www.ncbi.nlm.nih.gov/geo/) and analyzed using GEO2R (http://www.ncbi.nlm.nih.gov/geo/geo2r/) to assess the overlap of differential expressed genes in PBMC and T2D relevant tissues, and genes with Benjamini and Hochberg multiple-testing adjusted *p* < 0.05 were considered to be significant.

### Construction of the T2D interactome

We assembled all known interactions present in a human cell from available databases and literatures to build a human interactome^5^,^64^,^65^. In brief, our initial curated network contained information from high-throughput Yeast-2-Hybrid PPI, literature curated protein-protein interactions, metabolic enzyme-coupled interactions, protein complexes and kinase-substrate interactions. As discussed in the “Result” section, the existing human interactome does not capture the T2D modules. Besides, disease-causing genes tend to exhibit tissue-specific interactions^66^. Therefore, we integrated a *de novo* T2D co-expression network into the knowledge-based interactome to yield the T2Di. We applied the Algorithm for the Reconstruction of Accurate Cellular Networks (ARACNE) reverse-engineering algorithm and applied recommended parameters^67^ to our transcriptome data and generated a genome-wide T2D co-expression network using gene expression (FPKM) in a two-step manner^68^. We only selected robust interactions between gene-gene pairs with a stringent mutual information score of at least 1.

### An integrative approach to identify T2D interactome signatures

In complex diseases, disease-proteins tend to avoid hub genes. From an evolutionary perspective, disease-related mutations in topologically-central genes may cause severe impairment of development and tend to be deleted from the population. On the other hand, disease-related mutations in the topologically-peripheral regions of the interactome may improve adaptability and increase chance of viability^51^. Besides, genes coding for disease-related proteins tend to have higher probability of being connected to other disease proteins than non-disease proteins in the interactome^69^. Therefore, we made use of the DIAMOnD algorithm^11^, a type of Diffusion-based method, to select genes with significant fraction of their interactions with known T2D-disease genes discovered by all GWAS studies deposited in GWAS Catalogue (T2D-GWAS genes). As demonstrated by Ghiassian *et al*.^11^, 200 iterations of DIAMoND would be optimal to yield important seed pathways at a rate similar to the one within the seed proteins themselves and significantly higher than random expectation across 70 real diseases, including Type 2 Diabetes that we are studying. We thus employed 200 iterations as our starting point. DIAMOnD penalizes the scoring of hubs that interact with many known T2D-disease genes, since such high scores might be spurious due to high degree of connectivity of these hub genes.

Molecular perturbation of complex diseases usually affects expression of genes in modules^10^. Thus, besides the DIAMoND method, we used the jActiveModules algorithm, a type of Subnetwork-based method, to discover T2D-associated active hotspots from our expression data^70^. The algorithm calculates a network score, which is a weighted average of the z-scores of the individual network members. We used the recommended parameter values to invoke the algorithm [*Simulated Annealing: Start Temp*: 1.0, *End Temp*: 0.01, *Iterations*: 10^6^. *Regional Scoring* was disabled. *Number of putative modules to be detected*: 5, *Overlap Threshold*: 0.1]. The algorithm was run twice in succession, the first time aimed to identify high-scoring network(s) (HSN1) in T2Di, and the second time, a high-scoring subnetwork(s) within the initial high-scoring component(s). The output from the second run was taken as HSN2. Recursive application of the algorithm aimed to discover the most active sub-network(s) in HSN1 as previously described^70^. Since the full set of T2D disease genes is unknown, we cannot assess the performance of each respective algorithm directly in terms of true positives or negatives. In line with suggestion by Barabasi *et al*.^4^, we considered the experimentally derived biological expression data from actual disease cohort as the ultimate functional validation approach for potential disease associated genes. Thereby the DIAMOnD genes and the genes in HSN2 had to show differential expression in the T2D case-control cohort to be considered as T2Di signatures. The functions of the resultant disease module were analyzed using ClueGO v2.1.6^71^ with an adjusted *p* threshold for the pathway significance of 0.02. We used the Drug Gene Interaction Database (DGIdb)^72^ and the QIAGEN’s Ingenuity^®^ Pathway Analysis Release (June 2015) (IPA^®^, QIAGEN Redwood City, www.qiagen.com/ingenuity), based on the most robust experimentally-validated gene-gene relationships to assess the utility of these interactome signatures as anti-diabetic drug targets. The Chromatin interaction (Hi-C) data of K562 and GM06990 cell lines^73^ were analyzed using HiCNorm^40^ and Yaffe’s method^41^, and consensus interacting chromatin regions identified by both normalization methods in both cell lines were used.

### Quantitative PCR replication in an independent case-control cohort

Differential expression of the candidate genes in the T2Di was validated by quantitative real-time PCR (qRT-PCR). First strand cDNA was synthesized by the High-Capacity cDNA Reverse Transcription Kit (ABI Biosystems) using 0.5 μg of total RNA as template. SYBR Green qRT-PCR with SYBP^®^ *Premix Ex Taq*™ (Perfect Real Time, Takara) was run with 40 cycles on an ABI 7900HT Thermocycler. Expression levels were normalized to the expression level of β-actin. Mann-Whitney *U* test was used to test for differential expression of the genes. For genes with multiple transcripts, the common exons were used as targets for primer design and for real-time PCR. Primers used are listed in Table S15.

### Meta-analysis with the DIAGRAM Trans-Ancestry T2D study and regulatory properties of the interactome signatures

In the DIAGRAM (DIAbetes Genetics Replication And Meta-analysis) trans-ethnic T2D GWAS meta-analysis database (DIAGRAM-database)^42^, there were 26,488 cases and 89,964 controls recruited from multiple consortiums (DIAGRAM, AGEN-T2D, STA2D and MAT2D) with different ethnic groups including European, East Asian, South Asian, Mexican and Mexican American. We retrieved the association summary statistics, which have undergone three rounds of genomics control at the cohort level, after ethnic-specific meta-analysis and after trans-ethnic meta-analysis (http://diagram-consortium.org/downloads.html). The genomic inflation factor (λgc) was 1.05, suggesting minimal global inflation of test statistics with accounting for most of the population stratification. We referred all T2D-associated genes deposited in GWAS Catalogue as T2D-GWAS genes and T2D genes reported by the DIAGRAM meta-analysis^42^ as DIAGRAM-genes.

We used a LD-based SNP binning tool, named LDsnpR^35^, to identify additional SNPs in the DIAGRAM-database which might be associated with T2D, targeted at the gene level. We assigned SNP marker information and *p*-values from the DIAGRAM-database to individual genes based on the chromosomal position and the LD profile of the SNP (positional- and LD-based-binning, respectively). During the process, a SNP was binned to a gene if it is physically located within the pre-defined boundaries of the gene, or in LD with another genotyped SNP that is physically located within these boundaries of the gene. Gene bin definitions were based on Human Ensemble release 66 (March 2012). The LD data was based on that of the Han Chinese in *Beijing*, China (CHB) sample from HapMap Phase II release 27. The pairwise LD threshold was set at r^2^ ≥ 0.8.

Regulatory properties of the variants were retrieved from RegulomeDB^74^. SNPs in LD with lead SNPs were retrieved from HaploReg v3^75^. We used FORGE v.1.1 (http://browser.1000genomes.org/Homo_sapiens/UserData/Forge), which utilizes DNAse1 hotspots from the Roadmap Epigenomics project, to analyze cell-type specific enrichment of regulatory elements consisting of variants in perfect LD (r^2^: 1 and D’: 1) with the T2Di signatures. eQTL data was retrieved from MuTHER^2^ and GTEx^3^. We used the Sherlock statistical framework^38^ to identify potential disease-associated genes by matching eQTL signals with GWAS associations. Expressional regulatory SNPs (eSNPs) could act together in *cis*- and *trans*- to regulate the expression of a T2D-associated gene. Sherlock utilized moderately associated cis- and trans- eSNPs and GWAS associations instead of solely focusing on eSNPs and GWAS results that met stringent cutoff criteria. By integrating the analysis from eQTL and T2D-GWAS signals, the causal nature of these signals could be elucidated. Using this approach, Sherlock first searched all eSNPs of each gene using the whole genome eQTL data. For each eSNP, Sherlock evaluated its association with T2D using the DIAGRAM GWAS data with three different scenarios: (1) if the eSNP of a specific gene was associated with T2D in GWAS, a positive score would be given; (2) if the eSNP of this gene was not associated with T2D, a negative score would be assigned; and (3) association with T2D without eSNPs did not alter the score. Thus, the total score of a gene increased with increasing number of SNPs associated with both T2D and expression. Finally, Sherlock identified gene-disease associations by matching genetic signatures of gene expression with disease-related traits. Statistical inference was performed using Bayes statistical framework and Bayes factor (BF, the probability of the observed data under a specific model) of each SNP was calculated separately. For each gene, Sherlock computed individual logarithm of BF (LBF) for each eSNP in the alignment, and the sum of these constituted the final LBF score for the gene. The value of the LBF score of a gene reflected the strength of evidence (i.e., a larger LBF represented higher probability that the gene was associated with the disease).

### Data Availability

The sequence data from this study have been submitted to the NCBI SRA (http://www.ncbi.nlm.nih.gov/sra) under the accession number **SRP026359**.

## Additional Information

**How to cite this article**: Li, J.-W. *et al*. Interactome-transcriptome analysis discovers signatures complementary to GWAS Loci of Type 2 Diabetes. *Sci. Rep.* **6**, 35228; doi: 10.1038/srep35228 (2016).

## Supplementary Material

Supplementary Information

Supplementary Table S1

Supplementary Table S2

Supplementary Table S3

Supplementary Table S4

Supplementary Table S5

Supplementary Table S6

Supplementary Table S7
