# Supplementary material for: Interactome-transcriptome analysis discovers signatures complementary to GWAS Loci of Type 2 Diabetes
Source: Sci Rep. 2016 Oct 18;6:35228. doi: 10.1038/srep35228 (PMC5067504; doi:10.1038/srep35228)
Supplement: Supplementary Information [file srep35228-s1.pdf]

Supplementary material

Interactome-transcriptome analysis discovers signatures complementary to GWAS Loci of Type 2 Diabetes

Authors and Affiliations

Jing-Woei Li[1,2], Heung-Man Lee[3,4,5], Ying Wang[3,4], Amy Hin-Yan Tong[4], Kevin Y. Yip[1,5,6], Stephen Kwok-Wing Tsui[1,2,5], Si Lok[4], Risa Ozaki[3,4,5], Andrea O Luk[3,4,5], Alice P. S. Kong[3,4,5], Wing-Yee So[3,4,5], Ronald C.W. Ma[3,4,5], Juliana C.N. Chan[3,4,5]*, Ting-Fung Chan[1,5,6]*

[1]Hong Kong Bioinformatics Centre, School of Life Sciences, The Chinese University of Hong Kong, Shatin, N.T., Hong Kong
[2]Division of Genomics and Bioinformatics, School of Biomedical Sciences, The Chinese University of Hong Kong, Shatin, N.T., Hong Kong
[3]Department of Medicine and Therapeutics, The Chinese University of Hong Kong, Prince of Wales Hospital, Shatin, N.T., Hong Kong
[4]Li Ka Shing Institute of Health Science, The Chinese University of Hong Kong, Prince of Wales Hospital, Shatin, N.T., Hong Kong
[5]Hong Kong Institute of Diabetes and Obesity, The Chinese University of Hong Kong, Prince of Wales Hospital, Shatin, N.T., Hong Kong
[6]Department of Computer Science and Engineering, The Chinese University of Hong Kong, Shatin, N.T., Hong Kong

E-mail address:
Jing-Woei Li: marcowanger@gmail.com
Heung-Man Lee: leehman@gmail.com
Ying Wang: wying@gmail.com
Amy Hin Yan Tong: amyhytong@gmail.com
Kevin Y. Yip: kevinyip@cse.cuhk.edu.hk
Stephen Kwok-Wing Tsui: kwtsui@cuhk.edu.hk
Si Lok: lok1313@icloud.com
Risa Ozaki: risaozaki@cuhk.edu.hk
Andrea O Luk: andrealuk@cuhk.edu.hk
Alice P. S. Kong: alicekong@cuhk.edu.hk
Wing Yee So: wingyeeso@cuhk.edu.hk
Ronald C.W. Ma: rcwma@cuhk.edu.hk
Juliana C.N. Chan: jchan@cuhk.edu.hk
Ting-Fung Chan: tf.chan@cuhk.edu.hk

*To whom correspondence should be addressed
Ting-Fung Chan, School of Life Sciences, The Chinese University of Hong Kong, Shatin, NT, Hong Kong
Phone: (+852) 3943-6876; Fax: (+852) 2603-7246; Email: tf.chan@cuhk.edu.hk
Juliana C.N. Chan, Department of Medicine and Therapeutics, The Chinese University of Hong Kong, Prince of Wales Hospital, Hong Kong
Phone: (+852) 2632-3138; Fax: (+852) 2632-3108; Email: jchan@cuhk.edu.hk

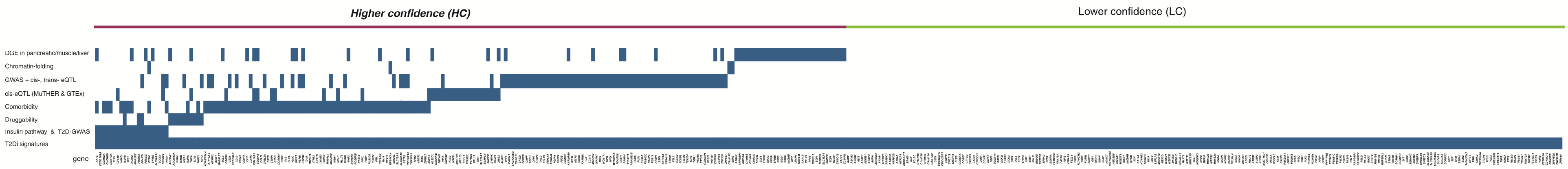

Figure S1: Summary of genomics and functional properties for all T2D interactome signatures. T2D interactome signatures overlapping with various genomics and functional properties are defined as follows: "DGE in T2D pancreatic/muscle/liver" indicates the signatures were also dysregulated in T2D relevant tissues, in addition to our discovery cohort; "Chromatin-folding" indicates the genes which may be distantly regulated by the GWAS SNPs reaching genome-wide significance in DIAGRAM-database; "GWAS + cis-, trans-eQTL" refers to the genes identified by the Sherlock statistical framework to be associated to T2D with the use of DIAGRAM-database, cis- and trans- eQTL signals; "cis-eQTL" refers to the SNPs in perfect LD to T2D interactome signatures with eQTL properties regulating these genes; "Comorbidity" refers to genes that are shared between T2D and comorbid diseases; "Druggability" indicates T2D druggable or potentially druggable targets; "Insulin & T2D-GWAS" refers to genes in the insulin pathway and T2D genes in GWAS Catalogue dysregulated in the T2D interactome signatures. The T2Di signatures overlapped with genomics and functional properties were considered to be higher confident set (HC), and those without were considered as the lower confidence set (LC).

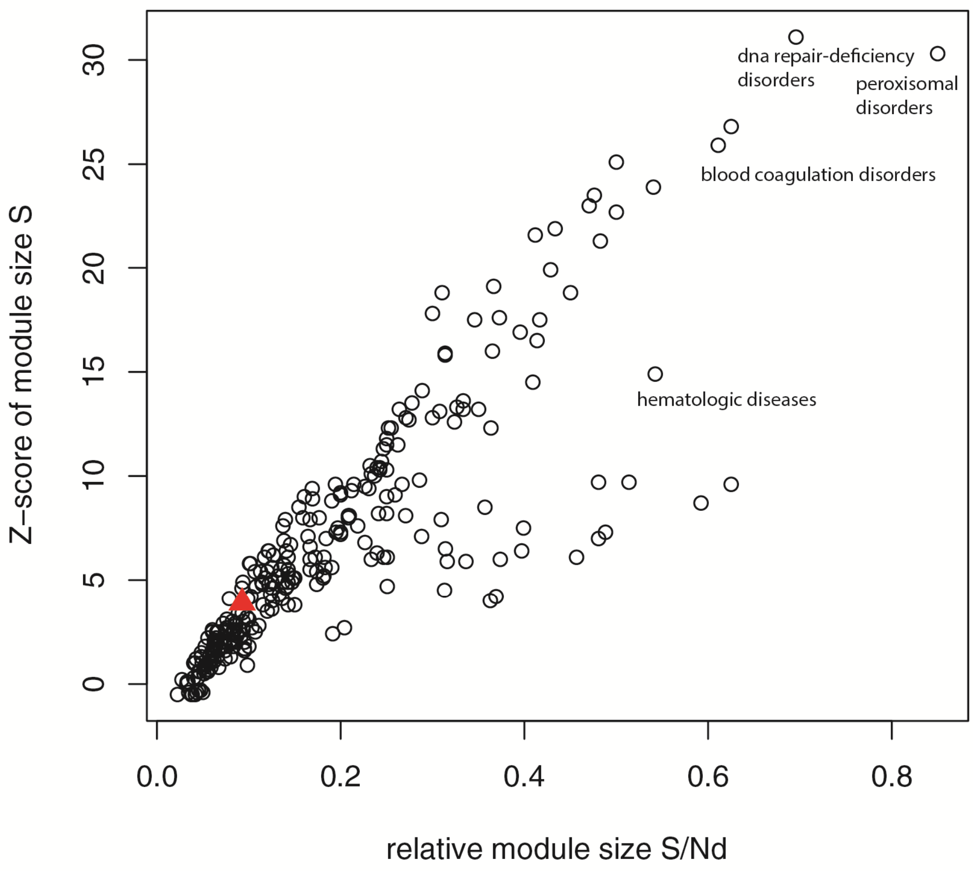

Figure S2: Statistical significance of the observed module as measured by z-score and relative module size S/Nd for all diseases. Red triangle indicates the T2D largest connected component consisting of 9 disease genes.

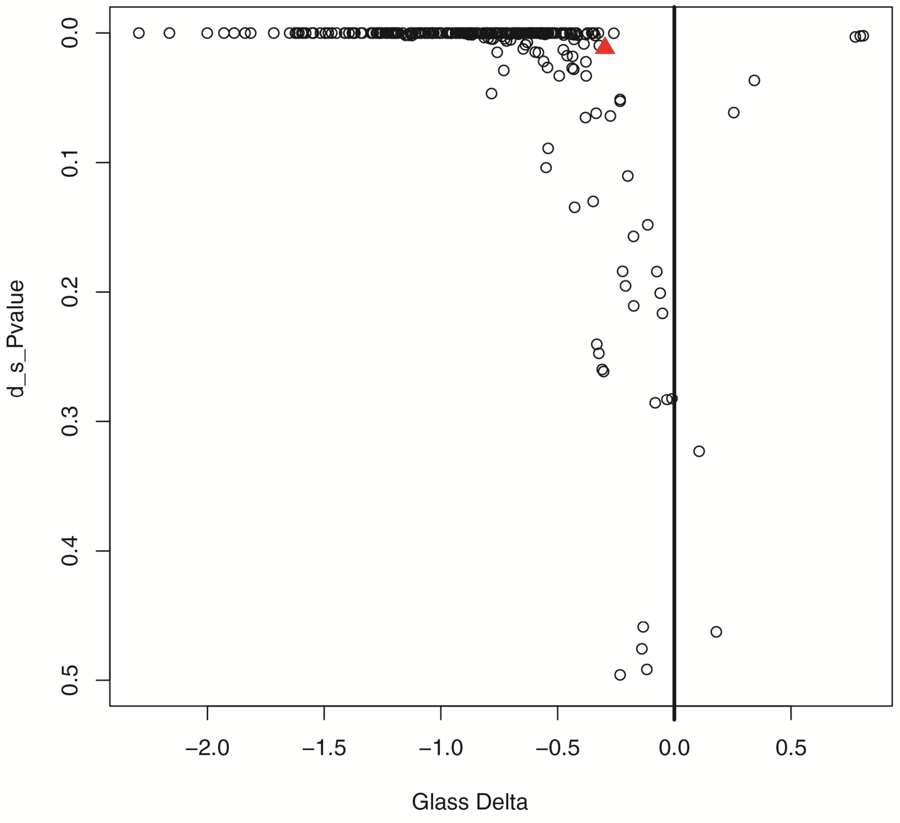

Figure S3 Statistical significance (*p*-value) and effect size (Glass' Delta) of the module localization as measured by the mean network-based distance for all genes in the diseases. Red triangle indicates the T2D largest connected component. Positive/negative Glass's Delta indicates the standard deviation larger/smaller than the mean of random expectation. Here, the combined evidence of Glass's Delta approaching zero (-0.297) and the low p-value (*p***:** 1.20E-2) suggested some T2D disease genes may not be localized to this largest connected component.

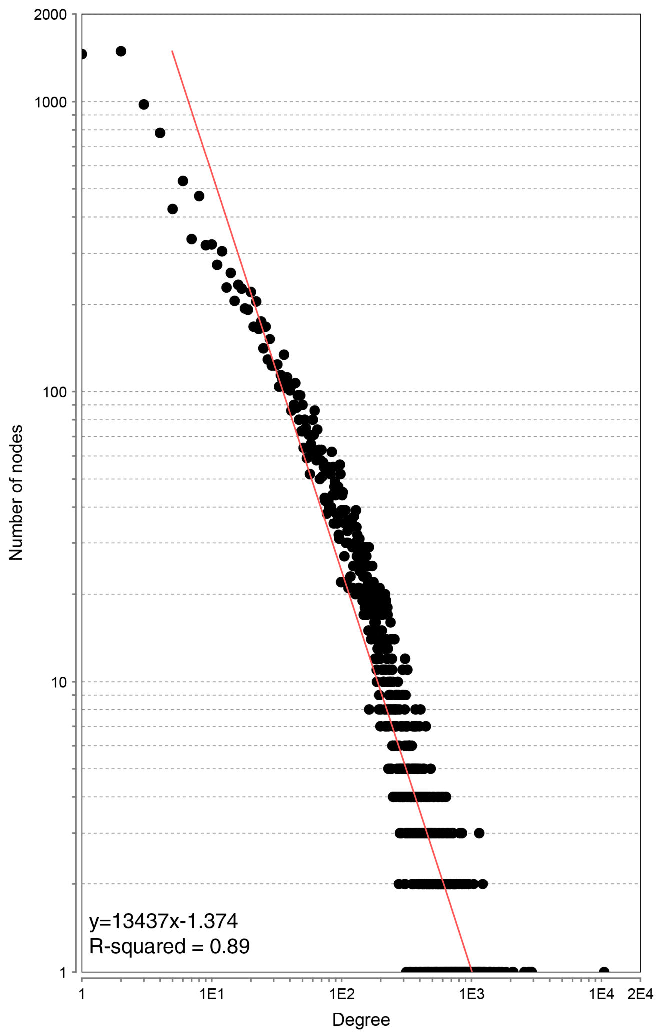

Figure S4: The degrees of interaction of the nodes in the T2D interactome follow a power law distribution.

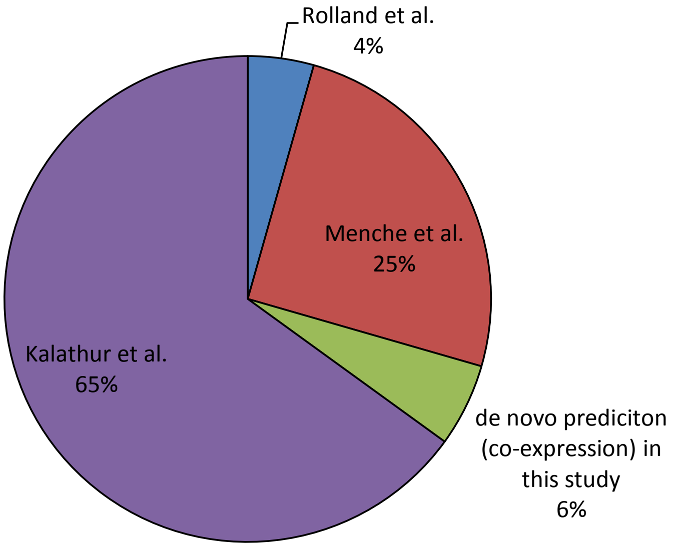

Figure S5. Distribution of interaction sources in T2D interactome. The gene network information of the T2Di were assembled from peer reviewed publicaitons shown in the references below, and also predicted using ARACHE based on co-expression patterns using RNA-Seq data generated in this study.

References

1. Menche, J. *et al.* Disease networks. Uncovering disease-disease relationships through the incomplete interactome. *Science* **347**, 1257601 (2015).
2. Kalathur, R.K. *et al.* UniHI 7: an enhanced database for retrieval and interactive analysis of human molecular interaction networks. *Nucleic Acids Res* **42**, D408-14 (2014).
3. Rolland, T. *et al.* A proteome-scale map of the human interactome network. *Cell* **159**, 1212-26 (2014).

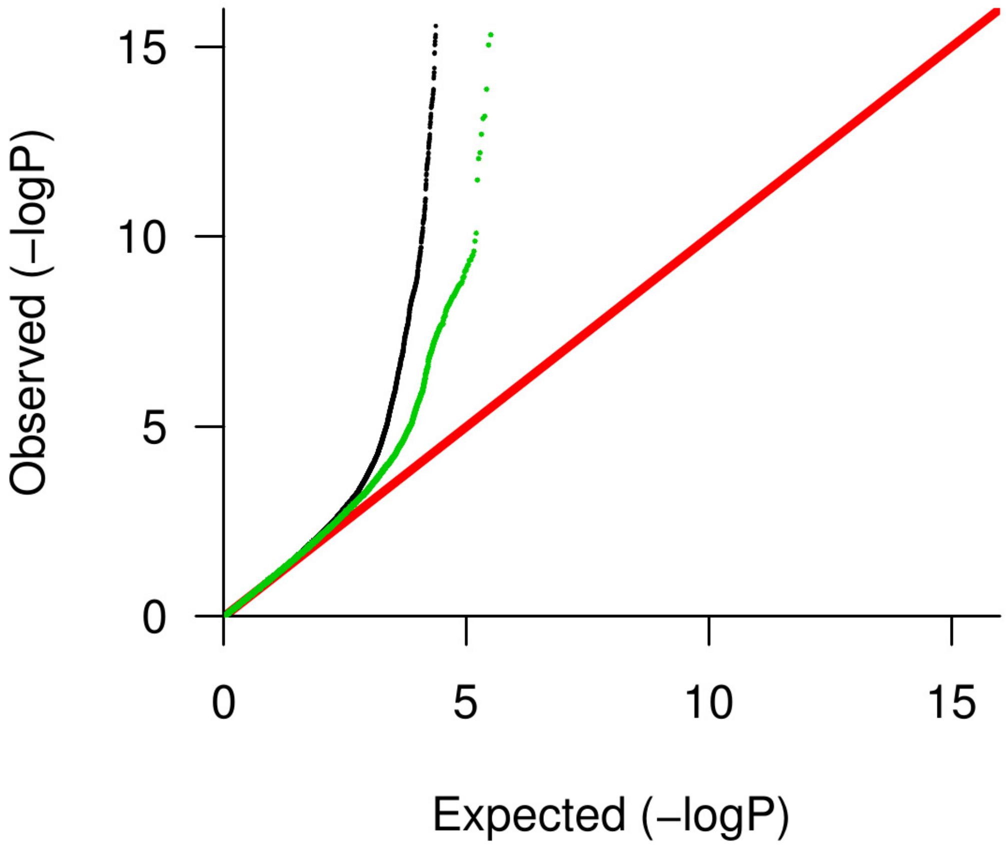

Figure S6. Quantile-Quantile (QQ) plot of all associations in DIAGRAM meta-GWAS (black curve), and association signals after removing all known T2D disease loci (green curve).

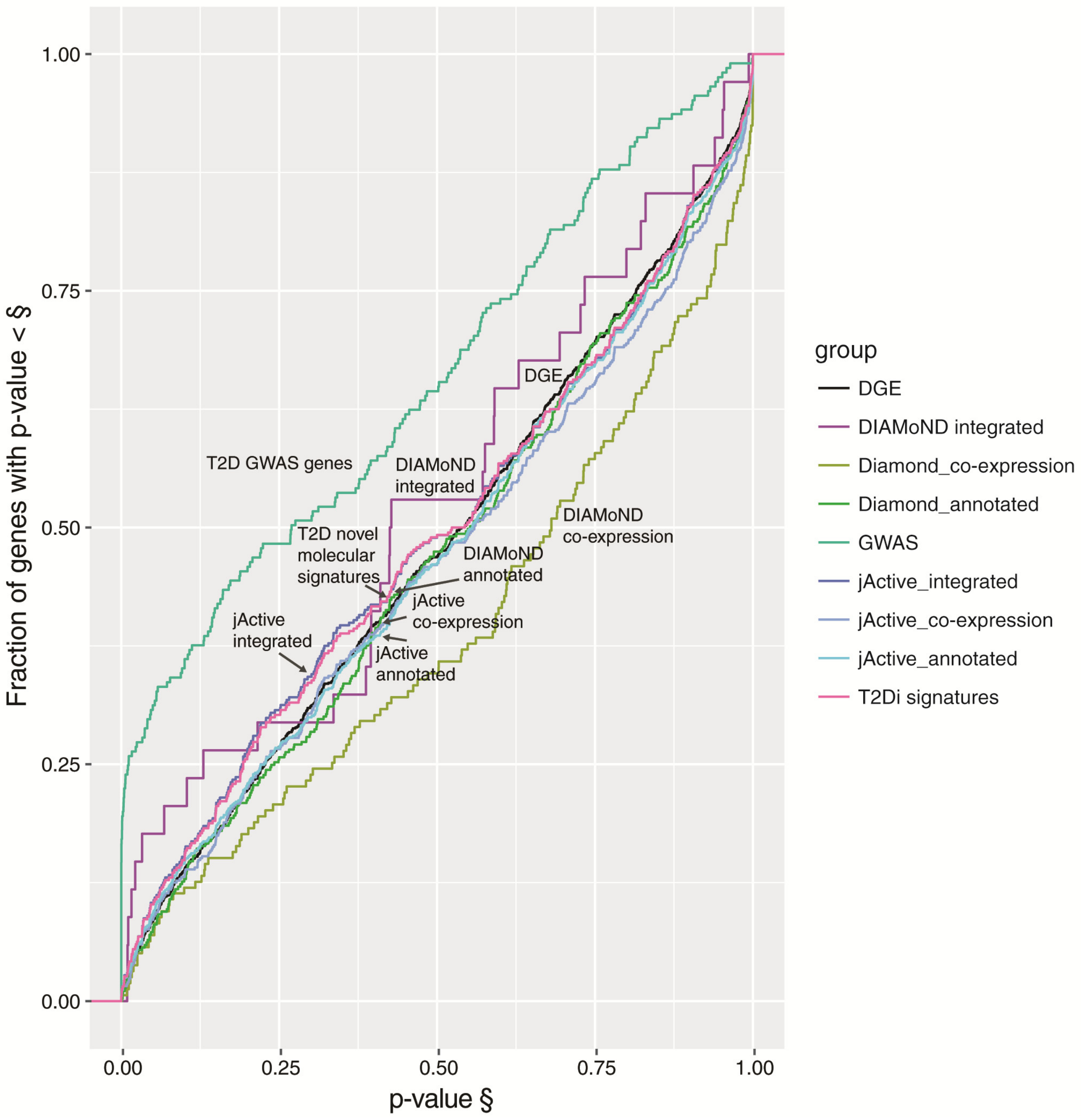

Figure S7. Comparison of disease genes identified by DIAMoND and jActiveModules in co-expression network (predicted by ARACHE in this study), annotated physical interaction network (from publications) and integrated network (constructed in this study).

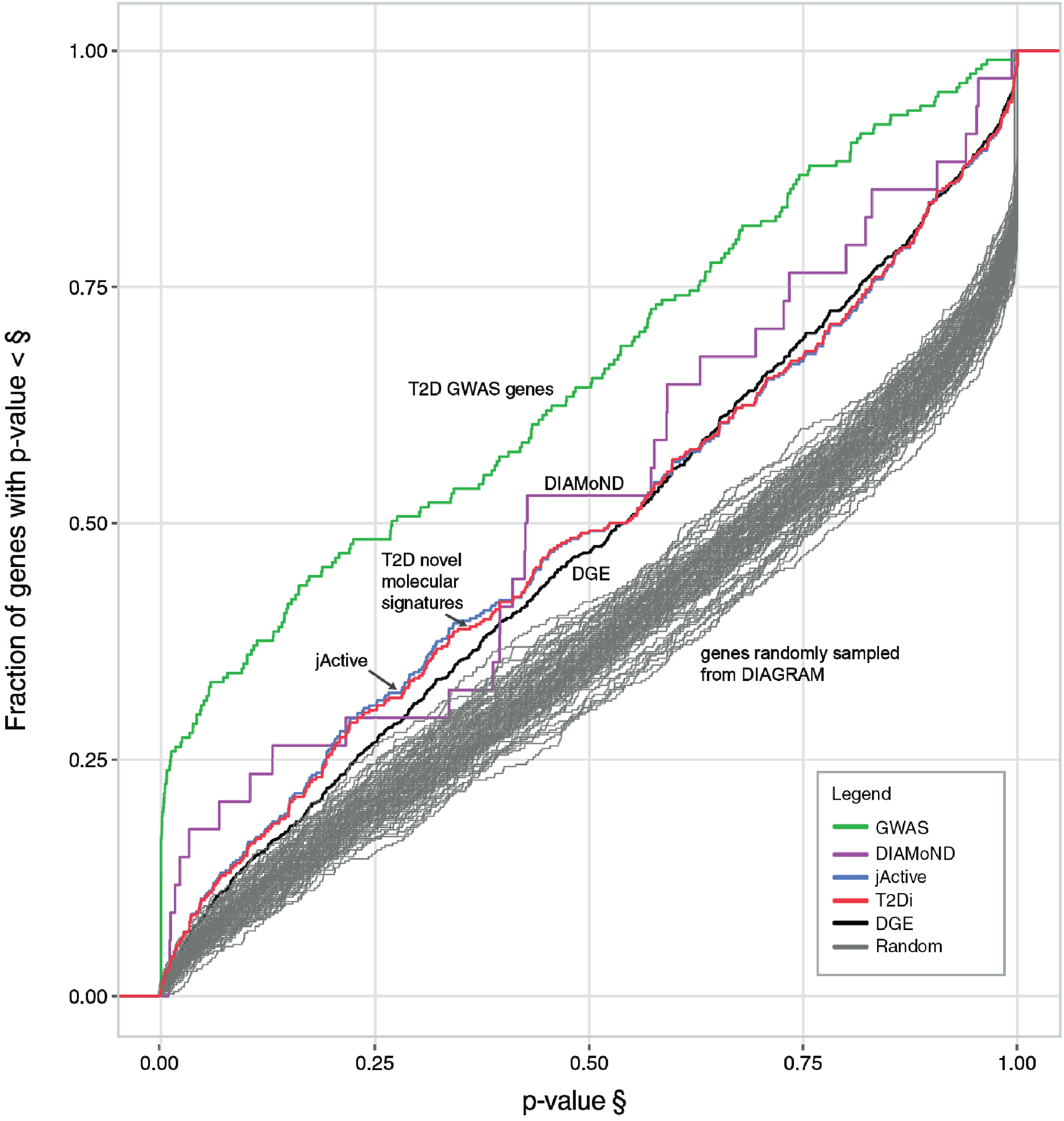

Figure S8. Association of genes to T2D discovered by DIAMoND, jActive, T2Di signatures (combining DIAMoND and jActiveModules) and differentially expressed genes generated in this study. Both DIAMoND, jActive and T2Di signatures were discovered using the integrated interactome**.** Association to T2D was assessed with respect to external & independent trans-ethnic GWAS data (DIAGRAM). Association to T2D of established T2D GWAS genes and 100 random gene sets were shown as comparison.

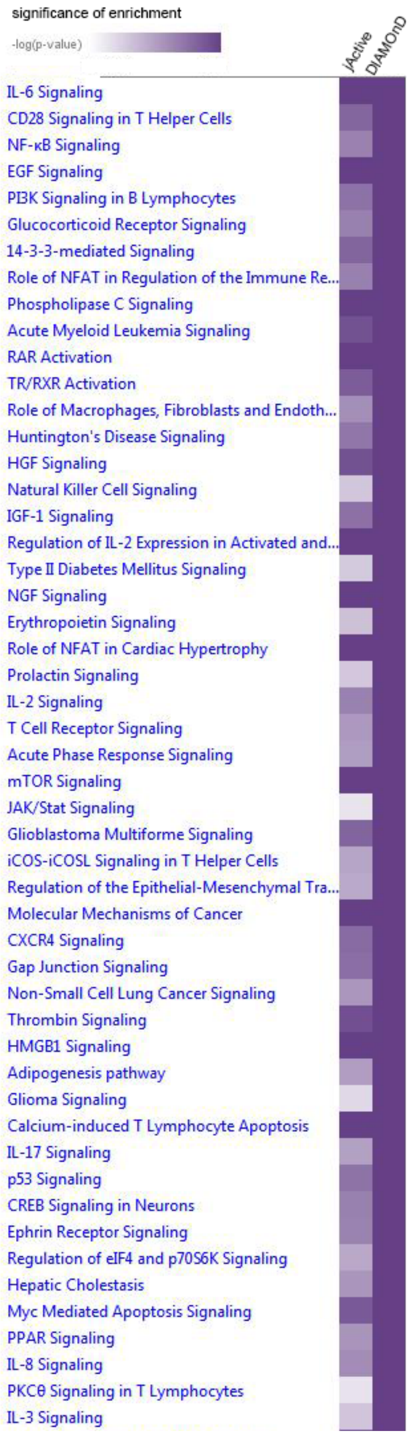

Figure S9. DIAMoND and jActiveModules identified different set of genes (DIAMoND identified 39 genes whereas jActiveModules identified 401 molecular signatures. Both method identified 20 common genes. The functional cohesiveness among genes discovered by DIAMoND and jActiveModules in T2Di signatures could first been demonstrated in the similarity of pathways enriched from respective methods.

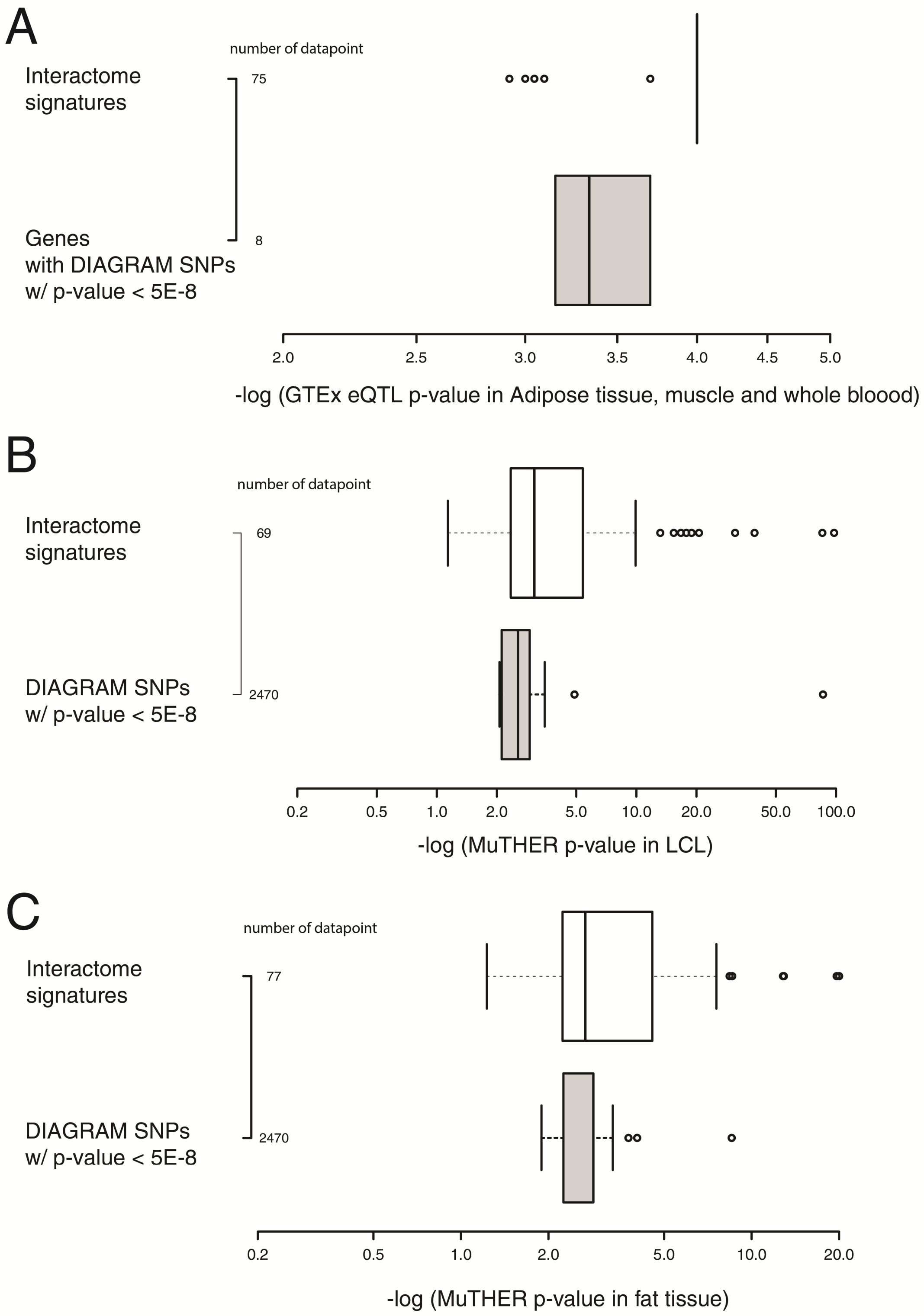

Figure S10. Comparison of eQTL p-values between T2Di signatures and DIAGRAM GWAS loci. T2Di signatures were compared to (**A**) GTEx adipose tissue, muscle and whole blood; (**B**) MuTHER LCL and (**C**) MuTHER fat tissues.

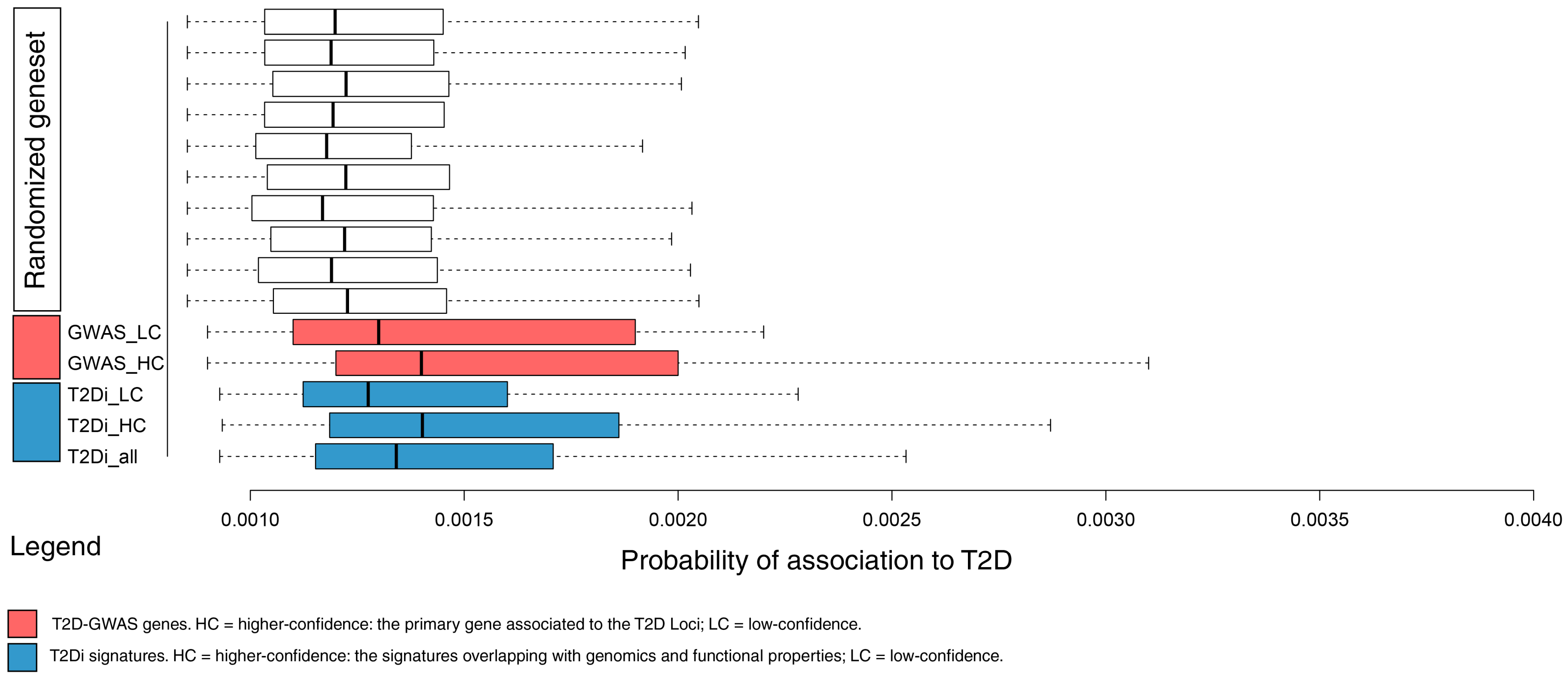

Figure S11. Comparison of T2D association probability between T2Di signatures, T2D-GWAS genes and randomized gene sets. The 10 set of randomized genes showed lower median probability association scores to T2D than the established GWAS genes and T2Di signature.

Supplementary Table 1: Sequencing summary and statistics

Sample ID	Status	Gender	Age	Raw reads (million)	Raw throughput (Gigabases)	Trimmed throughput (Gigabases)	Quality Trimmed % Mapped*
ZN991	Control	M	54	59	4.5	4.0	89.20%
ZN993	Control	M	51	69	5.2	4.3	89.35%
ZN994	Control	F	57	63	4.7	4.2	89.45%
ZN995	Control	F	61	46	3.5	3.2	88.73%
ZN996	Control	M	57	69	5.2	4.5	89.43%
ZN1048	Control	F	36	59	4.5	4.0	89.60%
ZN1083	Control	F	36	69	5.3	4.5	91.23%
ZN1084	Control	M	60	69	5.3	4.4	92.55%
GS28	Control	F	51	71	5.4	4.6	93.19%
GS29	Control	M	79	63	4.8	4.3	94.14%
ZN1040	DM	M	57	71	5.4	4.6	91.08%
ZN1041	DM	M	57	72	5.5	4.5	91.20%
ZN1042	DM	F	64	72	5.5	4.6	91.69%
ZN1043	DM	F	33	73	5.5	4.7	91.12%
ZN1044	DM	M	55	70	5.3	4.6	90.76%
ZN1045	DM	F	63	70	5.3	4.3	90.87%
ZN1046	DM	M	64	68	5.1	4.6	90.65%
ZN1085	DM	F	58	66	5	4.4	91.81%
ZN1086	DM	M	47	69	5.2	4.6	91.24%
ZN1047	DM	F	30	62	4.7	4.3	91.46%

Supplementary Table 2: Available separately

Supplementary Table 3: Differential expression of T2D-dysregulated genes observed in PBMC from Chinese T2D patients

Gene	*Description*	*Insulin pathway*	*T2D GWAS candidate*	*Novel molecular signatures*	*Fold Change (log 2)*	*B&H adjusted p*
AKT2	v-akt murine thymoma viral oncogene homolog 2	✔	/	/	-0.78	4.42E-04
PRKCZ	protein kinase C, zeta	✔	/	/	-0.77	2.90E-02
IRS1	insulin receptor substrate 1	✔	✔	/	-0.98	3.93E-03
PIK3R2	phosphoinositide-3-kinase, regulatory subunit 2 (beta)	✔	/	/	-0.886	3.53E-04
PPP1R3F	protein phosphatase 1, regulatory (inhibitor) subunit 3F	✔	/	/	-1.082	5.63E-04
PYGM	phosphorylase, glycogen, muscle	✔	/	/	-0.966	1.93E-03
PRKAR1B	protein kinase, cAMP-dependent, regulatory, type I, beta	✔	/	/	-1.005	2.74E-04
SH2B2	SH2B adaptor protein 2	✔	/	/	-1.297	1.45E-05
TRIP10	thyroid hormone receptor interactor 10	✔	/	/	-0.859	1.12E-02
SREBF1	sterol regulatory element binding transcription factor 1	✔	/	/	-1.400	2.23E-11
BAD	BCL2-associated agonist of cell death	✔	/	/	-0.841	8.41E-03
HRAS	v-Ha-ras Harvey rat sarcoma viral oncogene homolog	✔	/	/	-0.982	7.17E-04
MAP2K2	mitogen-activated protein kinase kinase 2	✔	/	/	-0.939	2.56E-05
ELK1	ELK1, member of ETS oncogene family	✔	/	/	-0.762	3.37E-03
SOCS1	suppressor of cytokine signaling 1	✔	/	/	-1.733	1.58E-07
WFS1	Wolfram syndrome 1	/	✔	/	-0.88	2.92 E-03
KCNQ1	potassium voltage-gated channel, KQT-like subfamily, member 1	/	✔	/	-0.87	3.43E-04
CHCHD9	coiled-coil-helix-coiled-coil-helix domain containing 9	/	✔	/	-1.17	6.81E-05
CDKN2A	cyclin-dependent kinase inhibitor 2A	/	✔	/	-0.87	1.27 E-02
SLC16A11	solute carrier family 16, member 11 (monocarboxylic acid transporter 11)	/	✔	/	-1.62	5.23E-05
GPSM1	G-protein signaling modulator 1 [Source:HGNC Symbol;Acc:17858]	/	✔	/	-1.27	6.33E-04
CCDC102A	coiled-coil domain containing 102A	/	✔	/	-1.34	6.26E-06
IL2RB	interleukin 2 receptor, bet	/	✔	/	-0.86	8.41E-05
PPARD	peroxisome proliferator-activated receptor delta	/	✔	/	-0.94	3.78E-05
DNMT3A	DNA (cytosine-5-)-methyltransferase 3 alpha	/	✔	/	-0.86	1.43E-04

Abbreviations: **B&H adjusted p**: nominal p-value adjusted by Benjamini and Hochberg method for multiple testing

Supplementary Table 4: Up-regulation of immune related genes in T2D PBMC

Gene	Description	Fold up-regulated in T2D	*P*-value	B&H adjusted p-value
CAMP	cathelicidin antimicrobial peptide	4.84	2.25E-16	2.20E-13
CCL8	chemokine (C-C motif) ligand 8	3.91	1.71E-05	5.50E-04
CCR2	chemokine (C-C motif) receptor 2	1.47	3.04E-03	3.13E-02
CXCL10	chemokine (C-X-C motif) ligand 10	2.22	3.17E-04	5.50E-03
INHBA	inhibin, beta A	3.96	2.30E-06	1.11E-04
LTF	lactotransferrin	5.09	6.27E-25	2.02E-21
PPBP	pro-platelet basic protein (chemokine (C-X-C motif) ligand 7)	2.32	4.61E-09	6.56E-07
S100A10	S100 calcium binding protein A10	1.63	2.11E-04	4.05E-03
S100A12	S100 calcium binding protein A12	1.82	2.49E-05	7.54E-04
S100P	S100 calcium binding protein P	1.87	2.97E-03	3.08E-02
SELP	carboxyl ester lipase pseudogene	1.88	1.58E-04	3.20E-03
TLR1	toll-like receptor 1	1.60	7.90E-04	1.13E-02
TLR2	toll-like receptor 2	1.70	7.29E-05	1.75E-03
TLR4	toll-like receptor 4	1.44	4.88E-03	4.33E-02
TLR5	toll-like receptor 5	1.55	3.36E-03	3.34E-02
TLR6	toll-like receptor 6	1.61	2.71E-03	2.88E-02
TLR7	toll-like receptor 7	1.73	2.25E-04	4.24E-03
TLR8	toll-like receptor 8	1.77	2.07E-05	6.47E-04

Abbreviations**: B&H**: nominal p-value adjusted by Benjamini and Hochberg method for multiple testing

Supplementary Table 5: Measure of perturbation of biological pathway by SPIA

KEGG pathway	Total Accumulation of pertubation	*p-value*	Pathway Status
Circadian rhythm - mammal	7.695	5.00E-06	Activated
Cytokine-cytokine receptor interaction	13.788	2.00E-03	Activated
Measles	15.197	3.00E-03	Activated
Basal cell carcinoma	-17.686	3.00E-03	Inhibited
Toxoplasmosis	13.964	1.00E-02	Activated
Axon guidance	-15.423	1.00E-02	Inhibited
Olfactory transduction	20.129	1.00E-02	Activated
Dopaminergic synapse	-6.475	1.50E-02	Inhibited
Wnt signaling pathway	-10.315	2.40E-02	Inhibited
Chemokine signaling pathway	23.599	2.70E-02	Activated
Transcriptional misregulation in cancer	-1.372	3.30E-02	Inhibited

Supplementary Table 6: Available separately
Supplementary Table 7: Available separately
Supplementary Table 8: Available separately

Supplementary Table 9: DIAGRAM *p*-values of the interactome signature having rSNP/eQTL property

Interactome signatures	rSNP of the interactome signatures	eQTL* in GTEx	eQTL* in MuTHER LCL	eQTL* in MuTHER Fat
PFKL	0.024	0.024		
ALS2CL	0.0032	0.0053		
CARD9	0.0019	0.033		
COL18A1	0.0016			
CTDP1	0.0034	0.024		
H1FX	0.0031		0.0026	0.0026
KLC2	0.03			
LRRK2	0.001	0.0036		
MAP7D1	0.0083	0.036		
PHPT1	0.0057		0.0069	
RPL13	0.0042			
SFI1	0.0027			
SLC12A7	0.0046	0.0068		
SREBF1	0.00074	0.0035		
SYMPK	0.001			
CACNA1I	0.00069		0.0082	
COL6A1	0.00091			
CTSD	0.027			
FBXW5	0.0057			
GPSM1	0.0019			0.033
INPP5E	0.00053			
MAFK	0.000076			
MED15	0.034			
MKL1	0.0024			
POLR2L	0.03			
PTPN23	0.0019			
SNAPC4	0.002			
SNRPN	0.00093			
THRA	0.007			
TRAF2	0.0057			
ZGPAT	0.007			
ZMIZ2	0.025			

Note:

QTL in GTEx: DIAGRAM SNP + SNPs in perfect LD (r^2: 1 and D': 1) to interactome signatures were queried to GTEx eQTL data in skeletal muscle, whole blood and subcutaneous fat

* eQTL *p*-value <0.05

Supplementary Table 10: Available separately
Supplementary Table 11: Available separately
Supplementary Table 12: Available separately

Supplementary Table 13: Clinical parameters of the participating T2D patients and controls in the discovery cohort

	**T2D**	**Control**
Number of subjects	10	10
Sex (M/F)	5/5	5/5
Age	52.8 ± 12.3	54.2 ± 12.4
Age of T2DM onset	30.2 ± 8.1	n/a
Duration of T2DM	22.6 ± 11.8	n/a
BMI	31.5 ± 4.8	21.6 ± 1.8
SBP (mmHg)	136.5 ± 13.7	116.2 ± 12.6
DBP (mmHg)	82.8 ± 10.1	75.3 ± 11.7
Fasting glucose (mmol/l)	10.3 ± 4.8	5.8 ± 1.4
HbA1c	8.4 ± 2.3	n/a
Total cholesterol (mmol/l)	4.2 ± 0.6	5.1 ± 0.6
HDL-C (mmol/l)	1.2 ± 0.4	1.5 ± 0.4
LDL-C (mmol/l)	2.2 ± 0.6	3.1 ± 0.6
Triglycerides (mmol/l)	1.9 ± 1.0	1.1 ± 0.5

Abbreviations: **BMI**: body mass index, **SBP**: systolic blood pressure, **DBP**: diastolic blood pressure, **HbA1c**: hemoglobin A1c, **HDL-C**: high-density lipoprotein cholesterol, **LDL-C**: low-density lipoprotein cholesterol

Supplementary Table 14: Clinical parameters of the participating T2D patients and controls in the replication cohort

	**T2D**	**Control**
Number of subjects	66	67
Sex (M/F)	21/45	27/40
Age	51.1 ± 7.3	49.9 ± 9.6
BMI	29.9 ± 4.7	22.5 ± 2.4
SBP (mmHg)	135.6 ± 15.3	115.8 ± 12.0
DBP (mmHg)	82.4 ± 10.9	73.1 ± 11.0
Fasting glucose (mmol/l)	9.2 ± 4.6	4.7 ± 0.4
HbA1c	8.2 ± 1.9	n/a
Total cholesterol (mmol/l)	4.8 ± 0.9	5.2 ± 0.8
HDL-C (mmol/l)	1.3 ± 0.4	1.8 ± 0.4
LDL-C (mmol/l)	2.8 ± 0.8	3.1 ± 0.7
Triglycerides (mmol/l)	1.9 ± 2.0	0.9 ± 0.3

Abbreviations: **BMI**: body mass index, **SBP**: systolic blood pressure, **DBP**: diastolic blood pressure, **HbA1c**: hemoglobin A1c, **HDL-C**: high-density lipoprotein cholesterol, **LDL-C**: low-density lipoprotein cholesterol

Supplementary Table 15: Primer list for qPCR replication

Gene	***Forward Primer***	***Reverse Primer***	***Product size (bp)***
SLC12A9	*GCAAAACCAACGACAAGGAT*	*CTGTCGTCACCACTGTGCTT*	*184*
SRRT	*ACTTCATCCTGGCACTCACC*	*CCGAAGACATCAAGCACAGA*	*205*
SREBF1	*AGGTGGAGGACACACTGACC*	*CAGGACAGGCAGAGGAAGAC*	*241*
SRF	*ACGACCTTCAGCAAGAGGAA*	*GAGAGTCTGGCGAGTTGAGG*	*199*
MLST8	*GCACCGGAAACTGCTATGTC*	*ACGTCCTCCAGATCTTGCAC*	*180*
CEBPB	*GCACAGCGACGAGTACAAGA*	*AGCTGCTCCACCTTCTTCTG*	*153*
